# Use of alteplase continuous rate infusion, pentoxifylline, and cyproheptadine in association or not, in acute feline aortic thromboembolism: a study of nine cats

**DOI:** 10.3389/fvets.2025.1512649

**Published:** 2025-05-12

**Authors:** Christopher C. Ray, Jacob Wolf, Julien Guillaumin

**Affiliations:** ^1^Veterinary Teaching Hospital, Clinical Sciences, Colorado State University, Fort Collins, CO, United States; ^2^Small Animal Hospital, Small Animal Clinical Sciences, University of Florida, Gainesville, FL, United States

**Keywords:** tPA, rtPA, tissue plasminogen activator, arterial, thrombosis, reperfusion, acute kidney injury

## Abstract

**Introduction:**

Locomotion improvement without serious complications is the main goal of treatment for feline aortic thromboembolism (FATE). We aimed to describe the survival and functional recovery of cardiogenic FATE treated with tissue plasminogen activator (rtPA) continuous rate infusion (CRI) and/or pentoxifylline (PTX) and/ or cyproheptadine (CYP), and to identify non-survivor characteristics.

**Methods:**

This is a retrospective, bicentric case series. Inclusion criteria were cardiogenic FATE cats receiving any of the medications described. Proportions of locomotion recovery, survival to discharge, reperfusion injury (RI) and acute kidney injury (AKI) were described. Admission and outcome characteristics were compared between survivors and non-survivors.

**Results:**

Nine cats were identified, 8/9 (88.9%) with bilateral FATE. Median age was 8.2 years (5.3–12.5). Median weight was 5.3 kg (4.1–7.1). Admission rectal temperature, affected limb lactate, creatinine and potassium were 36.9°C (35.4–38.8), 14.2 mmol/L (7.1–18.8), 114.9 umol/L (53–185.6) and 3.7 mmol/L (3.5–44.), respectively. No significant differences were found between survivors and non-survivors for relevant admission characteristics. Seven (77.8%) cats received tPA-CRI, 7/9 (77.8%) cats received pentoxifylline and 5/9 (55.6%) cats received cyproheptadine. Three (33.3%) cats received monotherapy (two rtPA-CRI, one PTX), 2/9 (22.2%) cats received dual therapy (one rtPA-CRI/PTX, one PTX/ CYP) and 4/9 (44.4%) cats received triple therapy. All study cats (100%) had an improvement in locomotion, and 4/9 (44.4%) survived to discharge. Reperfusion injury and AKI were documented in 3/9 (33.3%) and 4/9 (44.4%) of cats, respectively. Non-survivors had a greater proportion of AKI than survivors (4/5 (80%) and 0/4 (0%), respectively). No other outcome characteristics were different between survivors and non-survivors.

**Discussion:**

rtPA CRI, PTX and/or CYP could be considered for treatment of FATE.

## 1 Introduction

Cardiogenic feline aortic thromboembolism (FATE), defined as the migration of a left atrial thrombus into systemic arteries, is a commonly encountered disease in cats. Bilateral FATE has a guarded prognosis with a 30–40% survival to discharge proportion in cats treated with conventional therapy (1–5). Previous studies have identified effective ways of preventing further embolization through the use of antiplatelet and anticoagulant medications (6–8). This has allowed some cats with cardiogenic FATE to have low re-embolization rates (three out of 18 (16.7%) in one study) and median survival times of 502 days, provided they survive to discharge (8). Therapy aimed at effectively and safely removing the clot while minimizing complications in the hospital, allowing survival to discharge, is therefore desirable.

The Consensus on the Rational Use of Antithrombotics in Veterinary Critical Care (CURATIVE) domain 6 guidelines, published to guide the use of thrombolytic therapy in dogs and cats, recently suggested that thrombolysis can be attempted in acute (i.e., <6 h) FATE, although no specific protocol was recommended, and that strong evidence-based recommendations are lacking (9). Alteplase, a recombinant form of tissue plasminogen activator (rtPA), is a frequently used thrombolytic. Alteplase has a half-life of less than 5 min and has minimal persistent effects on the body, making it ideal for constant-rate infusion (10). A commonly used thrombolytic protocol in FATE (11, 12) is based on acute ischemic stroke (AIS) protocols in people (13); although AIS differs from FATE in etiology, organ(s) affected the amount of ischemic tissue and complications. Acute ischemic stroke has been shown to be most effectively treated when rtPA is given within 4.5 h of the event (14, 15). It is licensed in people in North America for administration of 0.9 mg/kg, with 10% of this dose administered as an initial bolus followed by an infusion of the remaining dose over 1 h.^1^ Sudden aortic thromboembolism is rare in people, is most often seen in neonates associated with umbilical catheterization, and is most commonly treated with rtPA continuous rate infusion (rtPA-CRI) and thromboprophylaxis, although clear recommendation and treatment consensus is lacking (16–18). In veterinary medicine, rtPA-CRI in six FATE cats was described in 1987 abstract, reporting a three out of six (50%) survival rate (19).

Common complications seen during FATE treatment, regardless of the use of thrombolytics, include reperfusion injury (RI) and acute kidney injury (AKI) (11, 12). Reperfusion injury occurs during recanalization of the clot, with subsequent flooding of the body with cytotoxic and inflammatory mediators (20). AKI has historically been thought to be related to occlusion of the renal vessels with clots; other contributing factors may include the release of vasoconstrictive substances, a lack of collateral circulation, and dehydration (11, 21). These comorbidities can be life-threatening, and identifying therapy to minimize them alongside maximizing recanalization of the aorta represents a significant target of FATE treatment. The development of collateral circulation is especially important, as experimental data showed that complete ligation of the aorta does not induce clinical paralysis in cats, whereas the use of vasoactive substances in the distal aorta without a physical occlusion would result in paralysis and lack of perfusion in the pelvic limbs (21).

Pentoxifylline (PTX) and cyproheptadine (CYP) are drug candidates to help mitigate AKI and RI. Pentoxifylline is a methylxanthine class medication that may aid kidney perfusion and reduce inflammation (22, 23), and studies evaluating its use in human neonatal vasospasm and thrombosis have been promising (24). Cyproheptadine is a serotonin receptor inhibitor capable of reducing platelet aggregation (25), and may oppose the vasoconstrictive and platelet-aggregatory effects of serotonin in experimental FATE if given prior to a FATE event (26). The use of these medications has never been studied in naturally occurring FATE.

Our primary aim was to describe the survival and functional recovery outcomes of cats presenting with cardiogenic FATE and treated with rtPA-CRI and/or pentoxifylline and/or cyproheptadine. Our secondary aim was to identify non-survivor characteristics. We hypothesized that treatment with an rtPA-CRI and/or pentoxifylline and/or cyproheptadine would result in a similar proportion of return to limb function with a similar proportion of RI, AKI and survival compared to historical studies on FATE treatment.

## 2 Materials and methods

### 2.1 Case identification, inclusion and exclusion

Cats in this retrospective case series were identified using the clinician’s personal case log at the Colorado State University (CSU) James L. Voss Veterinary Teaching Hospital (JG) or an rtPA prescription pharmacy search at the University of Florida (UF) Small Animal Hospital (JW) emergency rooms. Ethical approval was not required by the Institutional Animal Care and Use Committee due to the retrospective nature of this study. Cats with a diagnosis of cardiogenic FATE that received treatment including at least one of rtPA-CRI, PTX or CYP were included. FATE was diagnosed based on clinical observations of any limb affected by the following five criteria: pale, cold, pulseless, painful, and lack of motor function (11, 12). Cardiogenic FATE was then diagnosed based on left-atrial to aorta ratio > 1.6:1 on a right parasternal short axis cardiac point-of-care ultrasound view or by complete echocardiography performed or supervised by a diplomate of the American College of Veterinary Internal Medicine (Cardiology) (27). Cases were excluded if they did not receive at least one of the three previously mentioned therapies.

### 2.2 Data collection

Data collected were as follows: demographic (age, sex, and breed); FATE characteristics (i.e., FATE limb location and time of event to admission); physiological characteristics (i.e., admission body weight/rectal temperature/pulse rate/respiratory rate/Doppler blood pressure); clinicopathological variables (i.e., admission lactate (non-affected limb, averaged affected limb, differential lactate), admission systemic blood glucose/creatinine/potassium/platelet count, and maximum creatinine); disease processes (i.e., type of cardiac disease and comorbidities); treatment (i.e., rtPA use and dose, admission time and FATE onset time to rtPA treatment time, PTX use and dose, CYP use and dose, other in-hospital medications, and pre-hospital medications); measured outcomes (i.e., reperfusion injury and timing, AKI and timing, locomotion improvement; survival, length of hospitalization); and euthanasia (yes/no). Any other pertinent details to each case were recorded in a qualitative manner. All data were stored in commercially available software.^2^

Reperfusion injury was defined as a rise in serum potassium over the reference range for the used biochemistry analyzer. AKI was defined as per the Risk, Injury, Failure, Loss, End-Stage Renal Failure (RIFLE) ‘injury’ score [i.e., a ≥ 2 fold (100%) increase in serum creatinine from baseline (admission) using point-of-care blood gas analyzers, as previously described (11, 12, 28, 29)]. This definition, away from the IRIS classification (30), was used to limit capturing AKI from furosemide use (31) and make AKI diagnosis more reflective of underlying disease, as recently published (11, 12). Locomotion improvement was defined as mild, moderate, or marked based on regain of motor function without autonomous ambulation; with slow and intermittent autonomous locomotion; and with strong ambulation, respectively. Locomotion improvement was established retrospectively and independently by two of the authors, and conflicts were resolved through consensus.

Clinicopathological variables were measured using ALB800 FLEX (CSU, UF) and ABL90 FLEX PLUS (CSU).^3^^,^^4^ Values out of the reference range were recorded as follows: respiratory rate ‘panting’ as 150 breaths per minute, ‘low’ temperature as 32.2°C (90°F), creatinine above the reference range as 1,503 umol/L (17 mg/dL).

### 2.3 Statistical analysis

Normality was assessed using the Shapiro–Wilk test. Descriptive statistics for the study group were reported as median (min–max) for all continuous data due to the small sample size. Categorical data were presented as frequencies and percentages.

Mann–Whitney *U* and Fisher’s exact tests were used to assess differences between survivors’ and non-survivor’ admission characteristics, cumulative rtPA dose and outcome characteristics. All tests were run two-sided where appropriate, with exact *p*-values reported. Significance was set at *p* ≤ 0.05. All statistics were performed in SPSS.^5^

## 3 Results

Nine cats were identified from November 2021 to August 2024, with 6/9 (66.7%) coming from CSU and 3/9 (33.3%) from UF (Table 1). The breeds in the study group were domestic, with various coat lengths [5/9 (55.6%)], Bengal [2/9 (22.2%)], and 1/9 (11.1%) each of Sphynx and Turkish Angora.

**Table 1:** Admission characteristics of nine study cats treated with a combination (or not) of alteplase constant rate infusion, pentoxifylline or cyproheptadine, and comparison of survivor and non-survivor characteristics.

Variable	All cats (*n* = 9)	Survivors (*n* = 4)	Non-survivors (*n* = 5)	*p*-value
Age (years)	8.2 (5.3–12.5)	6.2 (5.9–9)	102.2 (5.3–12.5)	0.286
Sex (*n*, %)	5 male (55.6%)	2 male (50%)	3 male (60%)	1.000
Weight (kg)	5.3 (4.1–7.1)	5.4 (4.1–6.2)	5.3 (4.8–7.1)	0.905
Cardiac disease (*n*, %)	5 HCM (55.6%)	2 HCM (50%)	3 HCM (60%)	1.000
FATE location (*n*, %)	8 bilateral pelvic (88.9%)	3 bilateral pelvic (75%)	5 bilateral pelvic (100%)	0.444
Time to admission (hours)	3.4 (0.3–7)	4.4 (1.8–7)	2.7 (0.3–5.8)	0.413
Onset to rtPA use (hours)	4.5 (2.2–14.3)	9.4 (4.5–14.3)	4 (2.2–7.5)	0.381
Admission to rtPA use (hours)	1.7 (0.6–6.8)	4.8 (2.8–6.8)	1.3 (0.6–1.9)	0.095
Rectal temperature (°C); [^o^F]	36.9 (35.4–38.8); [98.5 (95.8–101.8)]	37 (36.1–38.2); [98.6 (97–100.8)]	36.9 (35.4–38.8); [98.4 (95.8–101.8)]	1.000
Pulse rate (bpm)	220 (180–280)	208 (180–250)	240 (180–280)	0.730
Respiratory rate (rpm)	56 (30–150)	50 (40–72)	60 (30–150)	0.905
Doppler blood pressure (mmHg)	103 (60–170)	170 (170–170)	81.5 (60–103)	1.000
Admit blood glucose (mmol/L); [mg/dL]	9.3 (4.8–15.3); [167 (86–276)]	14.6 (6.6–15.3); [262.5 (119–276)]	8 (4.8–10); [145 (86–189)]	0.111
Admit non-affected lactate (mmol/L)	3.2 (1.1–10.9)	2.9 (1.1–4.4)	4.7 (2.4–10.9)	0.190
Admit affected lactate (mmol/L)	14.2 (7.1–18.8)	12.1 (7.1–15.5)	16.5 (14.2–18.8)	0.400
Admit creatinine (umol/L); [mg/dL]	114.9 (53–185.6); [1.3 (0.6–2.1)]	106.1 (53–185.6); [1.2 (0.6–2.1)]	123.8 (106.1–185.6); [1.4 (1.2–2.1)]	0.556
Admit potassium (mmol/L)	3.8 (3.5–4.4)	3.8 (3.5–4)	4.1 (3.6–4.4)	0.486
Admit platelets (x10^9)	104 (29–238)	104 (29–156)	103 (41–238)	0.886

HCM, hypertrophic cardiomyopathy; FATE, feline aortic thromboembolism; rtPA, recombinant tissue plasminogen activator (alteplase).

One (11.1%) cat had a unilateral left forelimb embolism. Two (22.2%) cats had previously been diagnosed with heart disease (one restrictive cardiomyopathy phenotype and one undiagnosed bi-atrial enlargement). Three (33.3%) cats had non-cardiac comorbidity: exercise intolerance, mixed hepatopathy and chronic enteropathy/exocrine pancreatic insufficiency, and lower airway disease. Seven (77.8%) cats had at least one pre-hospital medication, as follows: methadone [3/9 (33.3%)], furosemide [2/9 (22.2%)], and 1/9 (11.1%) each of clopidogrel, ketamine, buprenorphine, gabapentin, diphenhydramine, pancreatic enzymes, and cobalamin. All pre-hospital medications were acutely prescribed by the referring veterinarian except one case each of furosemide, diphenhydramine, pancreatic enzymes, and cobalamin. Five (55.6%) cats were referrals, of which 4/5 (80%) were presented to CSU and 1/5 (20%) was presented to UF.

### 3.1 Treatments

Seven (77.8%) cats received rtPA-CRI, 7/9 (77.8%) cats received PTX and 5/9 (55.6%) cats received CYP (Table 2). Per individual patients, 3/9 (33.3%) cats received monotherapy: 2/9 (22.2%) rtPA-CRI and 1/9 (11.1%) PTX. One (11.1%) cat received rtPA/PTX dual therapy, 1/9 (11.1%) cat received CYP/PTX dual therapy, and 4/9 (44.4%) cats received rtPA/PTX/CYP triple therapy. The average PTX dose was 100 mg (range: 60–100 mg) every 12 h, with an average time to start PTX after admission of 14 h (range: 4–22 h). All cases that received CYP did so at a 2-mg dose every 12 h, with an average time to start CYP after admission of 12 h (range: 5–25 h). Specific rtPA doses used are shown in Table 3, with an average onset to rtPA administration of 4.5 h (range: 2.2–14.3 h). The average cumulative rtPA dose was 1.1 mg/kg (range: 1–2 mg/kg), and there was no difference in cumulative rtPA dose between survivors and non-survivors (*p* = 1.000).

**Table 2:** Treatment and outcome characteristics of individual study cats.

Cat number	Center	FATE Treatment	Locomotion improvement	Survived to discharge	LOH	RI	AKI
1	CSU	PTX	Mild	Yes	3.7	Yes	No
2	CSU	rtPA; PTX	Mild	No	1.3	No	No
3	CSU	rtPA; PTX; CYP	Moderate	Yes	2.4	No	No
4	CSU	rtPA; PTX; CYP	Marked	No	1.2	Yes	Yes
5	CSU	PTX; CYP	Moderate	Yes	2.7	No	No
6	CSU	rtPA; PTX; CYP	Mild	No	1.8	Yes	Yes
7	UF	rtPA	Moderate	Yes	3.0	No	No
8	UF	rtPA	Mild	No	1.0	No	Yes
9	UF	rtPA; PTX; CYP	Mild	No	2.8	No	Yes

CSU, Colorado State University; UF, University of Florida; FATE, feline aortic thromboembolism; PTX, pentoxifylline; rtPA, recombinant tissue plasminogen activator (alteplase); CYP, cyproheptadine; LOH, length of hospitalization; RI, reperfusion injury; AKI, acute kidney injury.

**Table 3:** rtPA-CRI dosing characteristics of seven cats suffering from cardiogenic FATE.

Total cumulative dose (mg/kg)	Dosing regime	Number (%); Cat number
1	0.1 mg/kg bolus then 0.1 mg/kg/h for 9 h	3/7 (42.9%); 2, 3, 4
1.1	0.1 mg/kg/h for 11 h (no bolus)	1/7 (14.3%); 8
1.6	0.16 mg/kg then 0.16 mg/kg/h for 9 h	1/7 (14.3%); 6
1.9	0.1 mg/kg/h for 19 h (no bolus)	1/7 (14.3%); 9
2	0.17 mg/kg for 12 h (no bolus)	1/7 (14.3%); 7

rtPA, recombinant tissue plasminogen activator (alteplase); CRI, continuous rate infusion; FATE, feline aortic thromboembolism.

All cats (100%) received thromboprophylaxis with clopidogrel and analgesia with a full *μ*-agonist during hospitalization. Seven cats (77.8%) received a 75 mg-loading dose of clopidogrel. Five (55.5%) cats received enoxaparin, and 3/9 (33.3%) different cats received rivaroxaban. Other in-hospital treatments included as follows: furosemide (*n* = 8); dextrose and maropitant (*n* = 5); regular insulin, calcium gluconate, lactated ringers, 0.45% sodium chloride and gabapentin (*n* = 4); potassium chloride, sodium bicarbonate, pimobendan, mirtazapine and n-acetylcysteine (*n* = 2); and one each of metoclopramide, ondansetron, spironolactone, buprenorphine, inhaled salbutamol, terbutaline, peritoneal dialysis, diltiazem, packed red blood cell transfusion, mannitol, hypertonic saline, potassium gluconate, s-adenosylmethionine, and pancreatic enzymes.

### 3.2 Outcomes

All (*n* = 9) cats had some degree of locomotor improvement, and 4/9 (44.4%) survived to discharge (Table 2). All non-survivors (*n* = 5) were euthanized. Admission characteristics between survivors and non-survivors were not different (Table 1). Three (33.3%) cats developed RI, and 4/9 (44.4%) cats developed an AKI. All cats that developed AKI had received furosemide. One cat was euthanized due to a repeat FATE event alongside a suspected cerebral ischemic event 24 h after stopping the rtPA-CRI and was the only case suspected of having a repeat thromboembolic event (1/9, 11.1%). This cat received 0.1-mg/kg/h rtPA-CRI for 19 h. The time from admission to development of RI and time from the first blood sample to AKI were 11 h (range: 7.6–14 h) and 12.3 h (range: 11–39 h), respectively. The degree of locomotor recovery was mild [5/9 (55.6%)], moderate [3/9 (33.3%)], and marked [1/9 (11.1%)]. The median length of hospitalization was 2.4 days (range: 1–3.7 days).

In order to investigate possible causes of euthanasia, comparisons of locomotion recovery, RI and AKI proportions between survivors and non-survivors were performed. There was a greater proportion of AKI in non-survivors (4/5 (80%)) than in survivors (0/5 (0%)) (*p* = 0.048). There was no difference in locomotion recovery (*p* = 0.087) or RI (*p* = 1.000) between survivors and non-survivors. The length of hospitalization (days) was significantly longer in survivors versus non-survivors (2.9 days (2.4–3.7) versus 1.3 days (1–2.8), *p* = 0.050).

## 4 Discussion

Our retrospective bicenter study described the treatment of a series of cats with cardiogenic FATE treated with rtPA-CRI and/or pentoxifylline and/or cyproheptadine. All cats in our case series regained motor function. Cats that did not survive were more likely to have an AKI.

Patient characteristics in our study were similar to previously reported FATE studies regarding age, weight, admission rectal temperature, heart rate, respiratory rate, hypertrophic cardiomyopathy (HCM) diagnosis, bilateral hindlimb FATE, and previously diagnosed heart disease (1, 2, 5, 7, 8, 12, 19, 32–34). Sex and breed characteristics in the study group were found to be 55.6% male and 50% domestic breed demographic. This differs from previous studies where males have typically been greatly over-represented and domestic breed frequency is over 60%; however, those differences are likely due to our small sample size.

Systemic blood glucose at admission in our study group was elevated, although lower than the largest prospective study in FATE cats (11). The significance of this finding is unclear. In dogs and people, hyperglycemia has been associated with worse outcomes, but not in cats (35–37). Hyperglycemia has been reported to be common in FATE cats (2); however, as in the general feline population, hyperglycemia has not been identified as a prognostic indicator in FATE. Admission blood glucose were not different between survivors and non-survivors in our study.

The novel rtPA protocol described in our study uses a CRI as performed in human neonatal aortic thromboembolism (16, 18), although the dosing (0.1–0.5 mg/kg/h, repeated if no effect), time frame (6-h infusion) and adjunct drugs (heparin; 10 U/kg/h) differ, and a CRI was chosen to try to optimize previously attempted treatment regimens (4, 11, 12). Studies on the use of rtPA in FATE have tested similar treatment protocols for people with AIS and failed to find benefits in survival or adverse outcomes compared to placebo; however, due to patient deaths, these studies became underpowered (11, 12). The most recent, a multicenter randomized control trial comparing a 1-h 1-mg/kg rtPA infusion to placebo failed to find a difference in AKI, RI or survival (11). Although non-significant, limb scores were consistently better in the rtPA group as was survival for 48 h. Type 2 error was likely a key factor in the lack of significant treatment effect found.

Our results are comparable to a previous clinical veterinary study, presented in an abstract form in 1987, that investigated the use of rtPA-CRI in cardiogenic FATE (19). Complications in the 1987 abstract were reported as being minor (limited to mild bleeding at the catheter site during rtPA infusion); however, the non-survivors [3/6 (50%)] were all reported to have died from reperfusion injury (defined as acidosis and/or hyperkalemia) (19). In a book chapter written by the same author, they mentioned that RI was not expected following FATE treatment and suggested it may have been due to rtPA use (38). That study enrolled six cats to receive rtPA-CRI without a placebo group, with an overall 50% (3/6) survival rate versus ours with 44% (4/9) survival and 100% (3/3) improvement in limb function in all surviving cats as we also found. Their study found a 50% (3/6) RI rate versus ours with a 33% (3/9) RI rate. Doses up to 8 mg/kg and timing between event and treatment up to approximately 30 h in the 1987 study may explain the higher RI rate in that study. Nevertheless, it provided the first evidence that rtPA may be successful in the treatment of cardiogenic FATE.

Another study (34) of 11 cats investigated two 5-mg/cat protocols, a 1.5-h rtPA infusion and a 4-h rtPA-CRI group, although the authors combined both protocols in the results. Among seven survivors 24 h after rtPA, 57% (4/7) of cats had motor function post-rtPA versus 28% (3/11) pre-rtPA. There was 64% (7/11) 24-h survival; however, only 27% (3/11) of the cats survived to discharge, and the study was terminated early due to high adverse events. This is compared to the 44% survival to discharge in our study. The high degree of adverse events was attributed to rtPA administration; however, the study did not include a control group. It should be noted that 36% (4/11) of the cats in that study received a second dose of the 5-mg/cat protocol, three of which died. This narrative of rtPA leading to poor outcomes (19, 34) is now being challenged, with a similar frequency of adverse events such as azotemia, hyperkalemia, and cardiac arrest in thromboprophylaxis-only protocols, and similar between rtPA group and control groups (11, 12).

The treatment modality in our study used an rtPA-CRI, as is reported for neonatal distal aortic thromboembolism (16, 18). Recommendation in neonatal medicine is an rtPA-CRI (0.1–0.5 mg/kg/h) for 6 h alongside a 10-U/kg/h heparin CRI in cases of major thrombosis, defined as absent femoral pulse alongside severe ischemia, defined as gangrene, tissue loss and/or paralysis (18). Moderate thrombosis, defined as absent femoral pulses and/or evidence of peripheral ischaemia (cool, pale, mottled extremities, and poor capillary refill time), is treated with rtPA-CRI if no improvement is noted with heparin only. If there is no clinical or radiological improvement after 6 h, a repeat infusion at the same dose and time frame is recommended (18). A surgical consultation for thrombectomy is also recommended at this later stage. However, evidence-based thrombolytic treatment in neonates is lacking (18). Future FATE studies may benefit from investigating neonatal rtPA protocols to improve outcomes.

Minimizing complications, such as AKI and RI, and promoting collateral circulation is paramount for treating clinical FATE. Experimental studies have shown that ligation of the distal aorta in normal cats does not create the clinical syndrome of FATE due to the development of collateral circulation. Instead, vasoactive substances injected in the distal aorta, even without physical obstruction, mimic the clinical syndrome of FATE (21, 26). The use of medications to promote collateral circulation has been suggested for more than 35 years, but our study is the first report of clinical use (38). Our study investigated the candidate drugs PTX and CYP based on the mitigation of vasoconstrictive effects of serotonin with CYP in the feline distal aorta (21, 26) and the vasodilatory effects of PTX (39, 40). One of the desired outcomes of using these medications is to improve functional recovery, and another desired outcome is to improve kidney perfusion, decrease the proportion of AKI, and decrease the proportion of RI.

Pentoxifylline is a methylxanthine drug that improves red blood cell rheology and induces vasodilation, and it has been shown to positively impact the restoration of blood flow in several experimental models (20, 40–43). It has been shown to maintain cerebral perfusion following ischemia in cats post-cardiac arrest compared to control without the drug if given pre-arrest, and it has some vasodilatory effects in feline cerebral and pulmonary vasculature that may mitigate renal arteriolar vasoconstriction (39, 40, 44). Cyproheptadine is a serotonin receptor inhibitor (26). Serotonin has been experimentally shown to have a role in vasoconstriction and the development of paralysis in the distal aorta of cats (21), and serotonin agonism has been shown to increase the risk of coronary stenosis and thrombosis in animal models (45, 46) and people (47). Cyproheptadine was an effective agent for preventing paresis or paralysis in an experimental model of FATE (i.e., double aortic ligation and injection of a mixture of cat blood and glass beads in the space created between the two ligatures) if given prior to the induced FATE event (26). Additionally, it has been shown to reduce serotonin-related vasospasm and platelet aggregation in the canine pulmonary vasculature (48) and may reduce serotonin-related vasospasm in human cerebral vasculature (49). Non-cyproheptadine serotonin antagonists have been shown to reduce coronary thrombosis in canine models when given prior to clot formation (50).

Our study is the first to report the use of PTX and CYP in clinical cardiogenic FATE and found locomotion improvement in all cats. Our study has higher locomotion recovery proportions than previous studies, although a direct comparison cannot be made (4, 11, 12, 34). The average cumulative rtPA doses in these studies and ours are similar, suggesting that the use of a CRI or the addition of PTX or CYP should be considered. Specifically, a 2022 abstract from France showed a significantly higher proportion of arterial recanalization (54.5% vs. 20.9%) and locomotion recovery (26.1% vs. 13.8%) with a 1-h, 1 mg/kg rtPA protocol (*n* = 52) than supportive care only (*n* = 65), with similar survival (35.5% vs. 34.7%) (4). This is an important consideration, as locomotion recovery is likely a major desired outcome in FATE. Locomotion recovery was not different between survivors and non-survivors in our study and may suggest reasons for euthanasia in this population are not related to locomotion recovery.

Compared to the most recent retrospective study on rtPA in FATE, our study found lower RI proportions [5/10 (50%) and 3/9 (33.3%), respectively] and higher AKI proportions [3/10 (30%) and 4/9 (44.4%), respectively] (12). The most recent prospective study on rtPA in FATE also found lower AKI rates (39%) but similar RI rates (33%) in our study (11). Further studies with a larger sample size are needed to investigate the impact of PTX or CYP or the use of rtPA as a CRI on RI and AKI. In our study, PTX and CYP were given more than 12 h after FATE onset and sometimes after starting rtPA. Major mechanisms for the development of RI are calcium overload, reactive oxygen species production, complement activation, and leukocyte migration (20). Flooding of the systemic circulation with these factors following occlusion relief leads to myocardial stunning and arrhythmia, and even once macroscopic blood flow is restored, an organ can continue to remain under-perfused (no-reflow phenomenon). Pentoxifylline, in particular, is an ideal candidate for reducing RI, as there is promising evidence for PTX’s ability to limit RI and hypoperfusion in experimental animal models if given before the RI event (40–42), including in a renal ischemia canine model (43) and hindlimb ischemia rabbit model (20).

Acute kidney injury was seen in 4/9 (44.4%) of the study population. This finding may indicate that thrombolysis and subsequent re-cannulation are associated with an increased risk of AKI; however, a small sample size limits the conclusion. It is unlikely that PTX and CYP contributed to the higher AKI rate based on their mechanisms of action. Our AKI rate is lower than that of a previously published study assessing AKI using IRIS criteria after furosemide use in cats with CHF (31). An altered definition of AKI, away from IRIS guidelines (30), was chosen in our study to limit the effect of furosemide use and may explain this difference; however, cases of AKI in our study likely still included furosemide-induced and transient AKI. Data are lacking regarding the incidence of AKI during treatment of any type for FATE. Many cats with FATE present with azotemia (2, 5), which differs from our study group, although low study numbers make this hard to interpret. Causes of AKI in FATE are likely multifactorial: thrombo-occlusive disease, mediators released as part of clot formation and subsequent vasospasm, and furosemide administration may all contribute (11). Serotonin release from platelet granules may be responsible for vasospasm (21). The presence of thrombi elsewhere in the body, such as pulmonary embolism, has been associated with AKI development in people (51). The rationale given for thrombotic-induced AKI is a mix of hemodynamic instability and renal congestion. Significantly more non-survivors developed AKI than survivors in the study group [4/5 (80%) vs. 0/4 (0%), respectively], and it is possible that greater study numbers would present a reduced survival benefit from AKI development in FATE. The use of thrombolytic therapy itself has also been associated with AKI development, specifically for the treatment of iliofemoral deep vein thrombosis in humans *via* catheter-directed thrombolysis; however, this risk has been attributed to other factors (bilateral and extensive thrombosis, single-session thrombolysis and female sex) rather than rtPA use itself (52). In contrast to this, similar AKI rates have been seen in FATE treatment both with and without rtPA use, and human AIS studies have failed to show thrombolysis as a risk factor for AKI (11, 12, 53).

Our study had 4/9 (44.4%) survivors and mirrors findings from prospective and retrospective studies that included a control group and failed to find a significant improvement in survival (11, 12). It is possible our study population represented a sicker group of cats compared to these previous studies: mean lactate concentration in the study group (14.2 mmol/L) was higher and above the previously identified cut-off associated with a worse outcome (11.5 mmol/L) (11). Although some of these findings can be due to the small sample size, they may represent a direct (through RI or AKI) or indirect (through survival bias) effect of thrombolysis treatment. This is illustrated by the fact that all non-survivor cats in our study group were euthanized despite having documented improvement in their locomotion. While finances may have played a role in the decision to euthanize, all non-survivors had a significantly shorter length of hospitalization than survivors, suggesting ethical and prognostic concerns may have been a primary motivator of euthanasia. A significantly lower proportion of survivors in the study group developed AKI versus non-survivors, a finding which may have led to a choice of euthanasia by clients. Future studies should document the reason for euthanasia in these cats to identify possible targets to reduce the high euthanasia rate, such as complications or perceived poor prognosis.

It is important to note the difference in survival compared to other studies using thrombolytic agents, such as streptokinase, rtPA, and urokinase; these studies had discharge rates similar to or lower than our study group (4/9 vs. 15/46, 3/11 and 5/12, respectively) (33, 34, 54). The more recently reported improved outcomes, both in the present study and others (4), may be influenced by improvement in standard treatment for FATE (7, 8), with some more recent evidence that in-hospital use of antithrombotics may aid in short-term clinical sign resolution (32). Finally, all but one cat in our study had bilateral hindlimb FATE, which has been associated with worse outcomes versus single limb FATE (33).

### 4.1 Limitations

Our study had a number of limitations. The lack of a control group and heterogeneous treatments means that conclusions that are drawn from this should be taken cautiously and should be considered hypothesis-generating. Bias may have been present in the dataset, given the data were collected over a 3-year period and at different institutions. One example is treatment bias, as the different centers involved in data collection may have various levels of experience treating FATE, which has been anecdotally associated with survival (2, 11). Clinicians were not blinded to the use of rtPA, which may have impacted their assessment of prognosis past the initial treatment phase based on motor improvement and pulse palpation. This may have led to unidentifiable effects of survival in each population. The sample size of our study group was low, likely increasing the chances of type 2 errors. Another very important limitation to consider is the difficulty in discerning treatment effect, as the cats in our study received a heterogenous treatment regimen. The rtPA dose in this study was non-standardized (as seen in the range of rtPA doses). There was also no standardization of medication administration, as not all cats received rtPA, PTX, and CYP (only three cases received all three medications). The pooling of these different treatment types limits the ability to make any conclusions about treatment effects. Furthermore, studies evaluating the use of adjunctive medications in FATE suggest that these medications must be given prior to ischemia and/or reperfusion, which was not the case in our study (26). Clopidogrel is reported to reduce platelet serotonin release and vascular smooth muscle response to serotonin (55, 56). Although all cats in our study received clopidogrel, not all were loaded, which may have impacted platelet kinetics and impacted the clinical course of these cats. A multicenter prospective approach would be ideal to maximize study numbers while standardizing treatments used and treatment timing (i.e., PTX and CYP prior to reperfusion) to allow conclusions on treatment effects to be made. A definition of AKI in our study differs from the IRIS guidelines, limiting the comparison to other studies, but was chosen to take into account the impact of furosemide on serum creatinine. Diagnosis of RI relied solely on the rise in serum potassium and does not account for other aspects of RI, such as reduced nitric oxide release and increased reactive oxygen species production, as well as increased creatine kinase, acidemia, arrhythmia and multiorgan dysfunction syndrome (20, 57, 58). However, diagnostic criteria for RI are not well defined in human or veterinary medicine, and potassium is a commonly used biomarker for RI (59, 60). Finally, the locomotion recovery assessment was subjective and based on retrospective data. The use of a subjective assessment makes it hard to accurately apply the results of this study and may have led to both type 1 and type 2 errors, depending on the assessor.

### 4.2 Conclusion

A combination of an rtPA CRI over 9 h and/or oral pentoxifylline and/or oral cyproheptadine may improve locomotion function in cats with cardiogenic FATE. Population numbers and data heterogeneity make any findings from this study difficult to interpret, and future studies should explore prospective evaluations of these medications alongside greater study numbers *via* a multicenter approach.

## Data Availability

The raw data supporting the conclusions of this article will be made available by the authors, without undue reservation.

## Footnotes

^1^ACTIVASE (alteplase) [package insert]. South San Francisco, CA 94080–4990: Genetech (2022).

^2^Microsoft Corporation, 2018. Microsoft Excel, RRID:SCR_016137. Available at: https://office.microsoft.com/excel.

^3^ABL800 FLEX, Radiometer Medical ApS, Bronshoj, Denmark, RRID:SCR_025871.

^4^ABL90 FLEX, Radiometer Medical ApS, Bronshoj, Denmark, RRID:SCR_025872.

^5^IBM Corp. Released 2022. IBM SPSS Statistics for Windows, Version 29.0. Armonk, NY: IBM Corp, RRID:SCR_016479.
